# Adverse Effects of Single Neutrophil Extracellular Trap-Derived Components on Bovine Sperm Function

**DOI:** 10.3390/ani12101308

**Published:** 2022-05-20

**Authors:** Claudia Moya, Rodrigo Rivera-Concha, Felipe Pezo, Pamela Uribe, Mabel Schulz, Raúl Sánchez, Carlos Hermosilla, Anja Taubert, Ulrich Gärtner, Fabiola Zambrano

**Affiliations:** 1 Center of Excellence in Translational Medicine—Scientific and Technological Bioresource Nucleus (CEMT—BIOREN), Faculty of Medicine, Universidad de La Frontera, Temuco 4780000, Chile; c.moya03@ufromail.cl (C.M.); r.rivera07@ufromail.cl (R.R.-C.); felipezo1982@gmail.com (F.P.); pamela.uribe@ufrontera.cl (P.U.); mabel.schulz@ufrontera.cl (M.S.); raul.sanchez@ufrontera.cl (R.S.); 2 Ph.D. Program in Medical Sciences, Faculty of Medicine, Universidad de La Frontera, Temuco 4780000, Chile; 3 Department of Internal Medicine, Faculty of Medicine, Universidad de La Frontera, Temuco 4780000, Chile; 4 Department of Preclinical Sciences, Faculty of Medicine, Universidad de La Frontera, Temuco 4780000, Chile; 5 Institute of Parasitology, Justus Liebig University Giessen, 35390 Giessen, Germany; carlos.r.hermosilla@vetmed.uni-giessen.de (C.H.); anja.taubert@vetmed.uni-giessen.de (A.T.); 6 Institute of Anatomy and Cell Biology, Justus Liebig University Giessen, 35390 Giessen, Germany; ulrich.gaertner@anatomie.med.uni-giessen.de

**Keywords:** bovine, spermatozoon, bull, neutrophil extracellular traps, membrane integrity

## Abstract

**Simple Summary:**

Neutrophil extracellular traps (NETs) play a key role in fertilisation by eliminating microorganisms and entrapping spermatozoa in the female reproductive tract. However, the individual exposure of bull spermatozoa to NET-derived components has not yet been explored. The aim of this study was to evaluate the individual effects of the main NET-derived proteins on sperm parameters. Sperm were selected and incubated for 4 h with different NET-derived proteins. Membrane and acrosome integrity, lipoperoxidation, and membrane phospholipid disorders were also evaluated. All NET-derived proteins/enzymes showed cytotoxic effects on bull sperm, and this effect should be considered in future investigations on the uterine microenvironment and advancement of spermatozoa in the female reproductive tract.

**Abstract:**

Neutrophil extracellular traps (NETs) play a key role in fertilisation by eliminating microorganisms and entrapping spermatozoa in the female reproductive tract (FRT). The deleterious effects of NETs on spermatozoa have been previously described; however, individual exposure to NET-derived components in bull spermatozoa has not been explored. The aim of this study was to evaluate the effects of the main NET-derived proteins, histone 2A (H2A), neutrophil elastase (ELA), myeloperoxidase (MPO), pentraxin 3 (PTX), cathepsin G (Cat-G), and cathelicidin LL37 (LL-37), at concentrations of 1, 10, and 30 μg/mL, on sperm parameters. Sperm were selected and incubated with different NET-derived proteins for 4 h. Membrane and acrosome integrity, lipoperoxidation, and membrane phospholipid disorders were also evaluated. Bovine polymorphonuclear neutrophil (PMN)/sperm co-cultures were evaluated by scanning electron microscopy and immunofluorescence. All NET-derived proteins/enzymes resulted in a reduction in membrane integrity, acrosome integrity, and lipoperoxidation at a concentration of 30 μg/mL. Bovine PMN/sperm co-cultures showed marked NET formation in the second hour. In conclusion, all NET-derived proteins/enzymes exerted cytotoxic effects on bull sperm, and this effect should be considered in future investigations on the uterine microenvironment and the advancement of spermatozoa in the FRT.

## 1. Introduction

Although remarkable scientific developments in the field of artificial insemination (AI) and the biology of reproduction in humans and domesticated animals have been achieved, infertility problems continue to increase worldwide [1]. The deposition of millions of spermatozoa into the female reproductive tract (FRT) results in early host innate immune reactions, such as the rapid infiltration of polymorphonuclear neutrophils (PMN) into the uterus. After insemination in mammals, PMN can remove excess sperm in vivo through phagocytosis to maintain a microenvironment favourable for embryo development. The first sperm is found in the oviduct minutes after mating, but these rapidly transported sperm cannot participate in fertilisation [2]. Sperm entering the oviducts do so within 8 h in ruminants, 1 to 2 h in pigs [3], and 0.5 to 4 h in horses [4]. Spermatozoa, seminal plasma, and semen extenders have roles in the induction of inflammation. In addition, the sperm number, concentration, viability, and site of semen deposition can modulate the inflammatory response. Cytokines, PMN, and mononuclear cells represent the uterine inflammatory response to mating [5].

PMN are the first cells of the innate immune system to migrate to any site of inflammation, acting in the elimination of invasive pathogens and in the pathogenesis of some autoimmune diseases, coagulopathies, and cancer progression/regression [6]. The effector mechanisms associated with PMN include phagocytosis, degranulation, cytokine/chemokine production, reactive oxygen species (ROS) production, and the production of neutrophil extracellular traps (NETs). NETs are DNA structures released into the extracellular space by protein arginine deiminase 4 (PAD-4)-mediated decondensation and the propagation of chromatin. Several proteins, enzymes, and peptides firmly adhere to extruded NETs fibres, including different types of histones (H1, H2A/H2B, H3, H4), and more than 30 primary and secondary granule components [7], including components with bactericidal activity, such as neutrophil elastase (ELA), myeloperoxidase (MPO), cathepsin G (Cat-G), lactoferrin, pentraxin 3 (PTX), gelatinase, proteinase 3 (PR3), cathelicidin LL37 (LL-37), peptidoglycan-binding proteins, and others, which can destroy pathogens and virulence factors [8]. At the reproductive level, studies have demonstrated that NETs have a deleterious effect on mammalian spermatozoa, which is especially related to the loss of motility and damage to the plasma membrane and acrosome in human and pig spermatozoa [9,10]. These recently described detrimental effects indicate the need to investigate the effect of proteins/peptides associated with NET structures on spermatozoa in more detail. Extracellular histones, one of the main components of NETs along with damage-associated molecular patterns, represent a good example of the role of NETs as a double-edged sword. Specifically, along with their other components, they have beneficial and detrimental effects, which not only protect hosts through bactericidal activities, but also exert cytotoxic effects on endothelial cells during sepsis or on insulin-producing beta cells in Type I diabetes [11]. It was also shown that NET components are cytotoxic to other types of cells in animals and humans [12]. NETs have a harmful effect on the function of male gametes in pigs and humans [10]; nevertheless, studies on bulls are still insufficient [1]. Since NETs have only been recently described, research has focused on their induction, microbicidal properties, and disease involvement. However, the functional consequences of NET interactions with other cells, such as spermatozoa, have not been assessed. Therefore, the aim of this study was to evaluate NET-derived proteins/peptides and enzymes individually, that is, ELA, MPO, Cat-G, H2A, PTX, and LL37, at different concentrations, to identify their specific cytotoxic effects on relevant sperm functional parameters in bulls.

## 2. Materials and Methods

### 2.1. Sample Acquisition and Ethical Declarations

Cryopreserved bull sperm samples were obtained from the purebred bull WINSTAR ALTAMAYAN-ET 011HO12288 (Minitube, Tiefenbach, Germany). All protocols were approved by the Scientific Ethics Committee of the Universidad de La Frontera with the authorisation code 120_20, and were conducted in compliance with Chilean Animal Protection Statute No. 20.380.

### 2.2. Study Site

The experiments were conducted in the Cellular Biology and Sperm Conservation Laboratory CEMT-BIOREN at the Universidad de La Frontera, Temuco, Chile.

### 2.3. Reagents

All reagents were acquired from Sigma-Aldrich (St. Louis, MO, USA), unless otherwise indicated.

### 2.4. Sperm Selection

Cryopreserved bull spermatozoa were used in this study. After thawing the straws, sperm were selected using a BoviPure^®^ density gradient, centrifuged at 600× *g* for 5 min, resuspended in 800 µL of Sp-Talp medium, and centrifuged again at 300× *g* for 4 min. The second washing step was performed at 300× *g* for 4 min, and the pellet was resuspended in 200 µL of sterile Sp-Talp medium.

### 2.5. Isolation of Bovine PMN

Blood sampling for PMN isolation was conducted using healthy adult dairy cows (*n* = 3) via jugular vein puncture. The heparinised blood was diluted in an equal volume of sterile phosphate-buffered saline (PBS), which contained 0.02% EDTA (Gibco, Dreieich, Germany). It was then layered on 12 mL of Biocoll^®^ separating solution (Biochrome AG, Berlin, Germany), and centrifuged (45 min, 800× *g*, room temperature (RT)). The upper layers containing plasma and peripheral blood mononuclear cells were eliminated, and the remaining sediment composed of PMN and erythrocytes was carefully isolated. The cellular sediment was gently re-suspended and shaken in 25 mL of sterile distilled water for 40 s to lyse erythrocytes. Osmolarity was adjusted immediately by adding 2.5 mL of sterile Hanks-balanced salt solution 10× (Biochrom AG). Isolated PMN were then washed twice (400× *g*, 10 min), resuspended in sterile RPMI 1640 cell culture medium, and counted in a Neubauer haemocytometer chamber according to Fichtner et al. (2020).

### 2.6. PMN/Sperm Co-Cultures and NET Visualisation via Scanning Electronic Microscopy (SEM) Analysis

Bovine PMN were incubated with selected sperm (as described in Section 2.4) at a 1:1 PMN:sperm ratio in sterile RPMI 1640 medium for 2 h (37 °C and 5% CO_2_ atmosphere) on a coverslip previously coated with poly-L-lysine (Sigma-Aldrich; 10 mm in diameter, Nunc, Dreieich, Germany). Next, the samples were fixed in 2.5% glutaraldehyde (Merck Darmstadt, Germany) for 15 min, and then post-fixed in 1% osmium tetroxide (Merck) for 1 h. After washing them in distilled water and dehydrating them, they were dried to a critical point via CO_2_ treatment and sprayed with gold. The samples were examined using a Philips XL30 SEM microscope at the Institute of Anatomy and Cell Biology of Justus Liebig University Giessen, Germany. 

### 2.7. Immunofluorescence for Neutrophil Elastase

Bovine PMN and spermatozoa (1:1) were incubated for 120 min at 37 °C in 5% CO_2_, fixed with 4% *p*-formaldehyde for 15 min, washed with PBS, and blocked in PBS supplemented with 2% BSA (PBS-BSA) for 30 min at RT. The samples were incubated overnight with a 1:300 dilution of rabbit anti-bovine ELA polyclonal IgG antibody (Abcam, Cambridge, UK) in PBS-BSA at RT, washed three times with PBS at 100 rpm of shaking for 5 min, and then incubated for 1 h with a 1:500 dilution of goat IgG anti-rabbit IgG secondary antibody conjugated with Alexa Fluor^TM^ 488 (Invitrogen, Thermo Fisher Scientific, Waltham, MA, USA) in PBS-2% BSA at RT. The samples were washed three times with PBS with 100 rpm in a Classic Orbital Shaker (DLAB, Beijing, China) for 5 min, and incubated with a 1:200 dilution of SYTOX© Orange (Invitrogen) for 15 min at RT for DNA staining. Finally, samples were washed with PBS and mounted with Fluoromount-G^TM^ mounting medium (Thermo Fisher Scientific, Waltham, MA, USA) for their visualisation via microscopy using a TissueFAXS i Plus Cytometry (TissueGnostics, Vienna, Austria).

### 2.8. Individual NET-Derived Component Exposure to Bovine Sperm 

In the experimental setting, three concentrations (1, 10, and 30 μg/mL) of the different NET-derived components, H2A, ELA, MPO, PTX, Cat-G, and LL37, were used. A sperm concentration of 750,000 vital spermatozoa suspended in 400 µL of sterile Sp-Talp medium was used to evaluate sperm parameters (i.e., membrane and acrosome integrity, lipid peroxidation, and changes in cholesterol membrane).

#### 2.8.1. Sperm Membrane Integrity 

This parameter was evaluated using an assay with SYBR14^®^ fluorescent probes and propidium iodide (PI). SYBR14^®^ (final concentration, 1 nM) and PI (final concentration, 5.8 µM) were added to 400 µL of the sperm suspension in Sp-Talp medium. The sperm suspension was incubated for 10 min at 38 °C, and centrifuged at 300× *g* for 5 min. The sperm pellet was resuspended in 200 µL of Sp–Talp medium for subsequent flow cytometric analysis (FACSCanto II flow cytometer, and FACSDivaTM software version 6.1.3 (Becton, Dickinson, and Company, Franklin Lakes, NJ, USA)), examining the percentage of live cells obtained according to Zambrano et al. [13]. 

#### 2.8.2. Sperm Acrosome Integrity 

This parameter was evaluated with an FITC-PNA/PI assay; FITC-PNA probe (final concentration 1 nM) and PI (final concentration 5.8 µM) were added to 400 µL of a sperm suspension in the Sp-Talp medium. The sperm suspension was incubated for 15 min at 38 °C, and centrifuged at 300× *g* for 5 min. The sperm pellet was resuspended in 200 µL of Sp-Talp medium for subsequent flow cytometric analysis. The results are expressed as the percentage of damaged acrosomes (PNA^+^, reacted acrosomes).

#### 2.8.3. Lipid Peroxidation 

In total, 5 µL of BODIPY-C11^®^ probe (stock solution 5 mM) and 0.5 µL of PI (stock solution, 5.8 µM) were added to 95 µL of the sperm suspension in Sp-Talp medium. The sperm suspension was incubated for 30 min at 38 °C, and centrifuged at 300× *g* for 5 min. The sperm pellet was re-suspended in 200 µL of Sp-Talp medium. Bodipy C-11 is a lipophilic probe that is incorporated into the sperm membrane in the midpiece and flagellum, and the oxidation of its polyunsaturated butadienyl portion, via interactions with peroxyl radicals, results in a change in the fluorescence emission peak from 590 to 510 nm (orange to green) [14]. A PI stain assay in combination with BODIPY C11 (base fluorescence, orange) has been performed; this staining changes to green when there is lipoperoxidation in the sample [14]. In the setup, PI fluorescence was isolated in the PerCP-Cy5-5 channel, to isolate the dead population and perform the analysis only on the live sperm population. For this, a PE minus 23% PerCP-Cy5-5 compensation was performed to isolate PI fluorescence and offset the original BODIPY C-11 fluorescence. Separating the living population from the dead sperm, the green fluorescence of the peroxidised sperm population was measured. The green arbitrary fluorescence units (A.U) of live sperm with BODIPY-C11 were determined by flow cytometry.

#### 2.8.4. Membrane Phospholipid Disorder 

Merocyanine 540 (MC-540) detects bicarbonate-induced changes in lipid packaging and distribution within the sperm plasma membrane, which are thought to be very early changes in the capacitation process [15]. This parameter was evaluated by performing the merocyanine/Sytox green assay. In brief, 400 µL of the sperm suspension in Sp-Talp medium was treated with 1 µL of MC-540 (1 mM) and 0.5 µL of Sytox green (20 µM) to stain dead cells. The sperm suspension was incubated for 30 min at 38 °C, and centrifuged at 300× *g* for 5 min. The sperm pellet was resuspended in 200 µL of Sp-Talp medium. This parameter was calculated using A.U of live sperm, and MC-540 was determined by flow cytometry. A setting with heparin 30 µg/mL (capacitated sperm) was performed as a positive control of the technique.

### 2.9. Flow Cytometry 

All fluorescence analyses were performed on a FACSCanto II flow cytometer (Becton, Dickinson and Company, BD Biosciences, San Jose, CA, USA), and samples were acquired and analysed using FACSDivaTM v. 6.1.3 (Becton). The sperm population was manually gated using the polygon gate option in a size-versus-internal complexity dot plot (forward scatter, FSC-A versus side scatter, SSC-A dot plot), which also included a FSC-A-versus-FSC-W dot plot to perform doublet discrimination (see Supplementary Figures). Positive and negative controls and the autofluorescence-unstained control were used to manually compensate for fluorochromes. The following compensation values were established: Sybr-14/PI; PE minus 30.0% FITC, PNA/PI; PE minus 18.94% FIT-C, BODIPY C-11/PI; PerCP-Cy5-5 minus 4.0% FITC, PE minus 23% PerCP-Cy5-5, MC-540/Sytox green; PE minus 37.50% FITC. Data from 10,000 sperm events were recorded in each experiment. The following lasers/filters were used for each technique: excitation of SYBR-14, PI, PNA, BODIPY C11, MC-540, and Sytox green was performed at 488 nm (blue) with an argon laser; FITC (530/30 nm, for SYBR-14, PNA, and Sytox green), PE (585/42 nm, for PI [combined with SYBR-14] and MC-540), PE (585/42 nm, for BODIPY), and PerCP-Cy5-5 (670LP, for PI combined with BODIPY C11).

### 2.10. Statistical Analyses 

Independent experiments were performed at least three times with different semen samples on different days and in duplicate. The results are presented as the mean ± standard deviation. Prism 9 software (GraphPad) was used for the statistical analysis. The D’Agostino–Pearson K2 test was used to evaluate the Gaussian distribution; when the numerical results did not pass the test of normality, they were transformed into arcsine values. One-way analysis of variance was performed using the Bonferroni test. Statistical significance was set at *p* < 0.05.

## 3. Results

### 3.1. Visualisation of Bovine Sperm-Induced NETs

**Figure 1:**
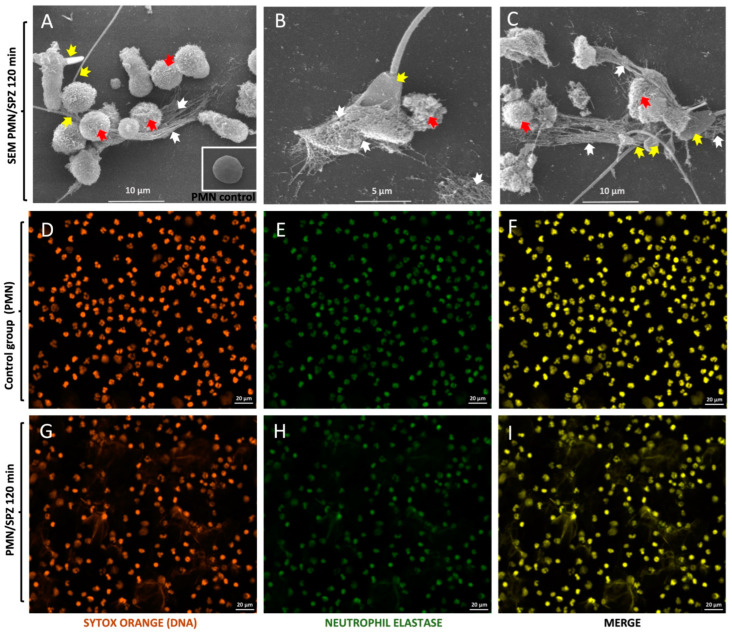
Visualisation of neutrophil extracellular traps (NETs) triggered by bovine spermatozoa using scanning electron microscopy (SEM) and immunofluorescence. Live bull sperm-induced NETs after 2 h of exposure to polymorphonuclear neutrophils (PMN) are shown. The SEM analysis and immunofluorescence of neutrophil elastase shows the activation of PMN in the presence of spermatozoa. The formation of NETs was triggered by spermatozoa, and spermatozoa were captured by NET structures. White arrows indicate NET structures, red arrows indicate PMN, and yellow arrows indicate sperm being trapped by NETs. Immunofluorescence images scale bars = 20 µm. SEM image scale bars = 5 and 10 µm.

SEM analysis confirmed that exposing PMN to viable bull sperm triggered the formation of NETs (Figure 1), which adhered firmly to the spermatozoa, apparently trapping them (Figure 1A–C). Spermatozoa were frequently captured by the tail or head (Figure 1A–C). In addition, it was demonstrated that when PMN were exposed to spermatozoa for 2 h, different levels of PMN activation were observed compared with those in the control group, which can be seen in the different pictures of Figure 1 (immunofluorescence pictures Figure 1D–I). Some PMN exhibited intact cell morphology (Figure 1A), whereas most were activated and underwent NETosis (Figure 1B,C). In addition, spermatozoa remaining trapped in a network of long, drawn-out fibres that originate from dead PMN are indicative of suicidal NETosis. Concerning NET phenotypes, the most frequently observed morphology corresponded to spread NETs (*spr*NETs) and diffuse NETs (*diff*NETs). Moreover, clusters of spermatozoa and fairly thick meshes of PMN-derived filaments, which can be seen more clearly in Figure 1A–C, were detected. Additionally, close contact between male gametes and the PMN surface was detected, whereas the others corresponded to phagocytosed spermatozoa.

### 3.2. Effects of H2A on Bovine Sperm Viability

**Figure 2:**
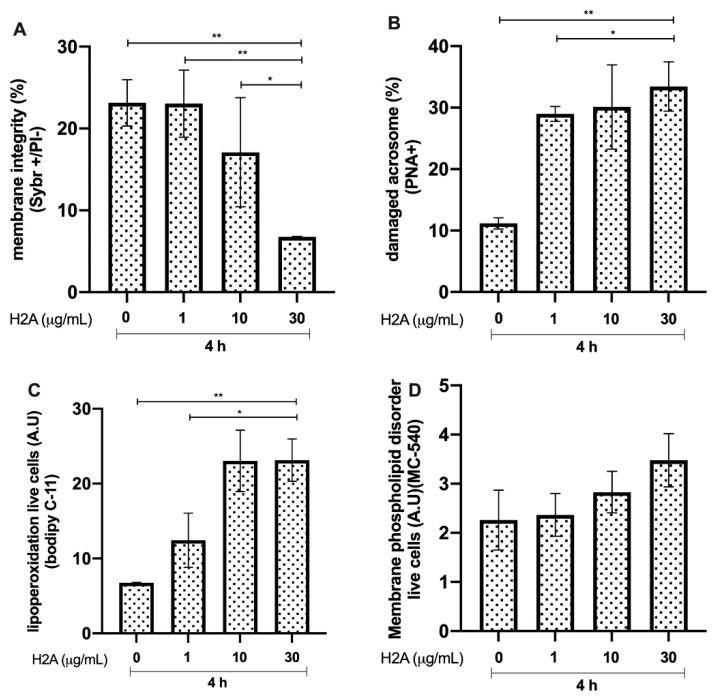
Effect of different concentrations of histone 2A (H2A) on bull sperm. (**A**) Plasma membrane integrity. (**B**) Damaged acrosomes. (**C**) Lipid peroxidation. (**D**) Membrane phospholipid disorder. The results are shown as the mean ± standard deviation. The asterisks (*, **) above the bars indicate differences between the groups (*p* < 0.05, and *p* < 0.01) based on three biological replicates.

Bovine sperm membrane integrity after 4 h of incubation with H2A showed a significant reduction in the group incubated with 30 μg/mL compared to that in the controls (*p* < 0.01, **). The group incubated with 30 μg/mL H2A showed lower membrane integrity than the group incubated with 1 μg/mL and 10 μg/mL H2A (*p* < 0.01, ** and *p* < 0.05, *, respectively, Figure 2A). Acrosome damage was significantly increased in the group incubated with 30 μg/mL, compared to that in the control group (*p* < 0.01, **). In addition, the group incubated with 1 μg/mL H2A showed higher acrosome integrity (*p* < 0.01, **) than the group incubated with 30 μg/mL H2A. In addition, the group exposed to an H2A concentration of 1 μg/mL showed a higher percentage of spermatozoa with intact acrosomes (*p* < 0.05, *) than the group incubated with 30 μg/mL of H2A (Figure 2B). The results of lipid peroxidation (Figure 2C) showed a significant increase in this parameter in the group incubated with 30 μg/mL, compared to that in the control group (*p* < 0.01, **). The group incubated with 30 μg/mL H2A showed a significant increase in lipoperoxidation compared with that in the group incubated with 1 μg/mL (*p* < 0.05, *). The membrane phospholipid disorder results did not show any significant changes between the experimental groups and the control group (Figure 2D).

### 3.3. Effects of MPO on Bull Sperm

**Figure 3:**
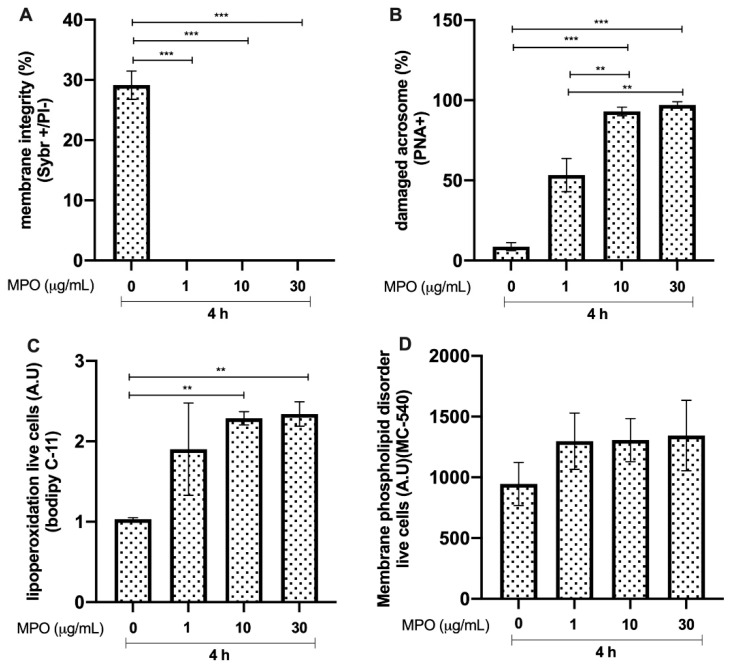
Effect of different concentrations of myeloperoxidase (MPO) on bull sperm. (**A**) Plasma membrane integrity. (**B**) Damaged acrosomes. (**C**) Lipid peroxidation. (**D**) Membrane phospholipid disorder. The results are shown as the mean ± standard deviation. The asterisks (**, ***) above the bars indicate differences between the groups (*p* < 0.01, and *p* < 0.0001) based on three biological replicates.

Bull sperm membrane integrity after 4 h of incubation with MPO showed a significant reduction (*p* < 0.0001, ***) in all of the experimental groups compared to that in the control group (Figure 3A). In addition, there was a significant increase (*p* < 0.0001, ***) in the percentage of spermatozoa with damaged acrosomes with concentrations of 10 and 30 μg/mL, compared with that in the control group without MPO. Furthermore, sperm incubated with 10 and 30 μg/mL of MPO showed a reduction in acrosome integrity, compared to that in the group treated with 1 μg/mL MPO (*p* < 0.01, **; see Figure 3B). The results concerning sperm lipid peroxidation showed an increase in this parameter, with 10 and 30 μg/mL of MPO compared with that in the control (*p* < 0.01, **; Figure 3C). The magnitude of membrane phospholipid disorder was not different compared to that in the control without MPO (Figure 3D).

### 3.4. Effects of ELA on Bull Sperm

**Figure 4:**
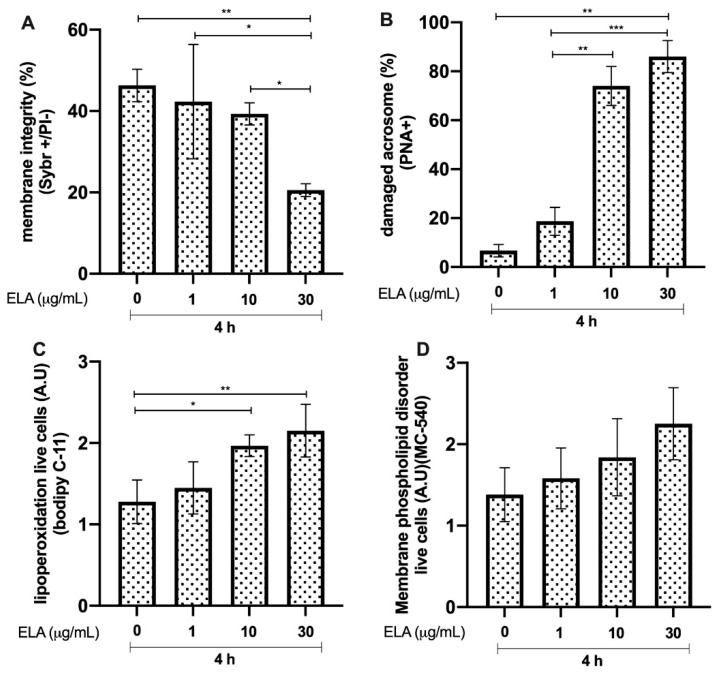
Effect of different concentrations of neutrophil elastase (ELA) on bull sperm. (**A**) Plasma membrane integrity. (**B**) Damaged acrosomes. (**C**) Lipid peroxidation. (**D**) Membrane phospholipid disorder. The results are shown as the mean ± standard deviation. The asterisks (*, **, ***) above the bars indicate differences between the groups (*p* < 0.05, *p* < 0.01, and *p* < 0.0001) based on three biological replicates.

Bull sperm membrane integrity after 4 h of incubation with ELA resulted in a significant reduction in the group exposed to 30 μg/mL, compared to that in the control group (*p* < 0.01, **). The group incubated with 1 μg/mL ELA showed greater membrane integrity (*p* < 0.05, *) than the group incubated with 30 μg/mL ELA. The group incubated with ELA at a concentration of 10 μg/mL showed greater viability (*p* < 0.05, *) than the group incubated with 30 μg/mL of ELA (Figure 4A). In addition, there was a significant reduction (*p* < 0.01, **) in the percentage of spermatozoa with intact acrosomes, with a concentration of 30 μg/mL compared to that in the control without ELA. Furthermore, the group incubated with 30 μg/mL of ELA showed a significant increase in acrosome damage, compared to that in the groups treated with 1 and 10 μg/mL of ELA (*p* < 0.01, ** and *p* < 0.0001, ***, respectively; Figure 4B). The results of lipid peroxidation showed a significant increase in the 10 and 30 μg/mL groups compared to that in the control (*p* < 0.05, * and *p* < 0.01, **, respectively; Figure 4C). The extent of membrane phospholipid disorder was not different compared to that in the control group without ELA (Figure 4D).

### 3.5. Effects of Cat-G on Bull Sperm

**Figure 5:**
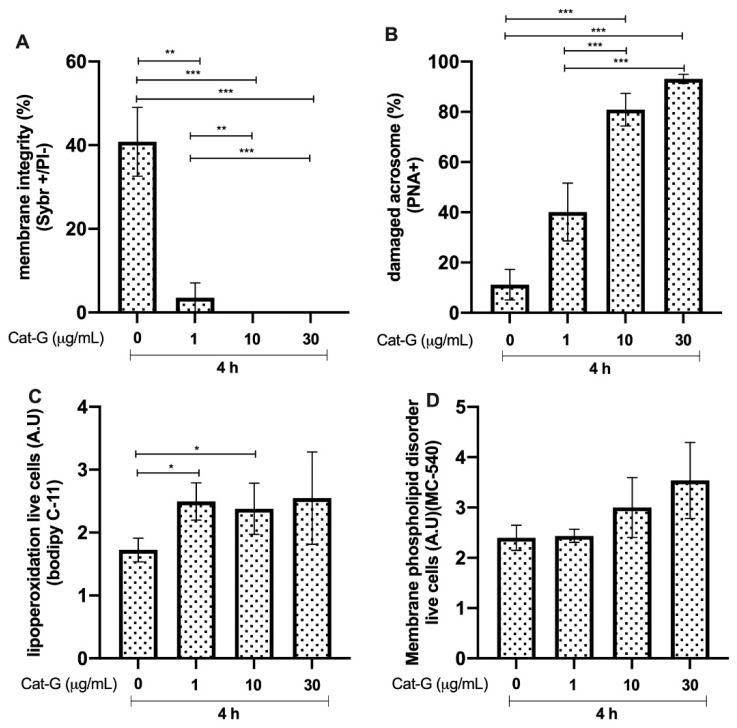
Effect of different concentrations of cathepsin G (Cat-G) on bull sperm. (**A**) Plasma membrane integrity. (**B**) Damaged acrosomes. (**C**) Lipid peroxidation. (**D**) Membrane phospholipid disorder. The results are shown as the mean ± standard deviation. The asterisks (*, **, ***) above the bars indicate differences between the groups (*p* < 0.05, *p* < 0.01, and *p* < 0.0001) based on three biological replicates.

Bull sperm membrane integrity after 4 h of incubation with Cat-G was significantly reduced in the groups incubated with 1 μg/mL (*p* < 0.01, **), 10 μg/mL (*p* < 0.0001, ***), and 30 μg/mL compared to that in the control group. The group incubated with 1 μg/mL showed a higher percentage of spermatozoa with higher membrane integrity than the group incubated with 10 and 30 μg/mL of Cat-G (*p* < 0.01, ** and *p* < 0.0001, ***, respectively; Figure 5A). The groups incubated with 10 and 30 μg/mL of Cat-G showed an increase in acrosome damage compared to that in the control group (*p* < 0.0001, ***; Figure 5B). The results of lipid peroxidation in live cells showed a higher A.U of BODIPY C-11 in the groups treated with 1 and 10 μg/mL of Cat-G. The extent of membrane phospholipid disorder was not different compared to that in the control group without CAT-G (Figure 5D).

### 3.6. Effects of LL-37 on Bull Sperm

**Figure 6:**
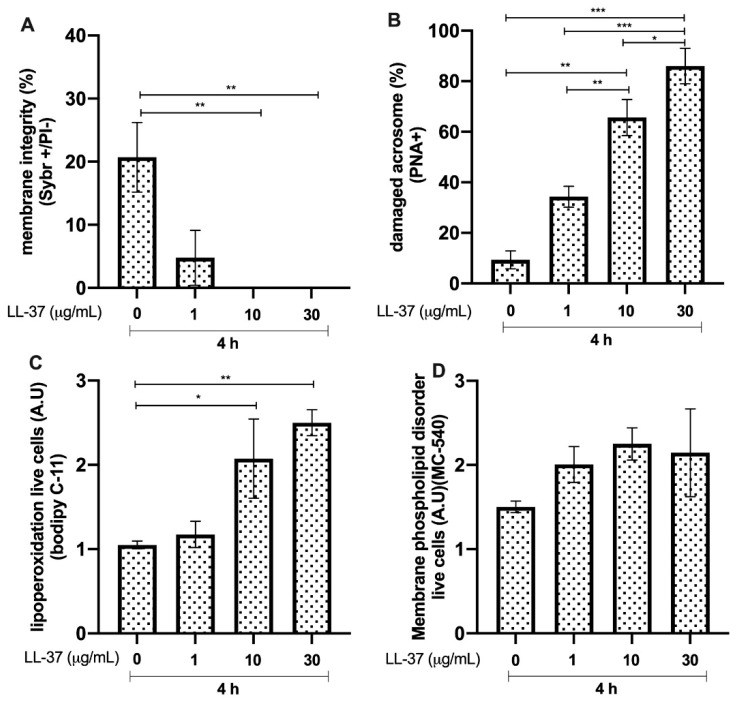
Effect of different concentrations of cathelicidin LL37 (LL-37) on bull sperm. (**A**) Plasma membrane integrity. (**B**) Damaged acrosomes. (**C**) Lipid peroxidation. (**D**) Membrane phospholipid disorder. The results are shown as the mean ± standard deviation. The asterisks (*, **, ***) above the bars indicate differences between the groups (*p* < 0.05, *p* < 0.01, and *p* < 0.0001) based on three biological replicates.

Bull sperm membrane integrity after 4 h of incubation with LL-37 was significantly reduced in the groups incubated with 10 and 30 μg/mL compared to that in the control (*p* < 0.01, **; Figure 6A). The groups incubated with 10 and 30 μg/mL of LL-37 showed an increase in acrosome damage, compared to that in the control group (*p* < 0.01, ** and *p* < 0.0001, ***, respectively). A concentration of 1 μg/mL of LL-37 resulted in a higher percentage of the sperm population with intact acrosomes compared to the groups treated with 10 and 30 μg/mL (*p* < 0.01, ** and *p* < 0.0001, ***, respectively). In addition, the 10 μg/mL group showed a higher percentage of spermatozoa with intact acrosomes than the 30 μg/mL group (*p* < 0.05, *; Figure 6B). The lipid peroxidation results showed increases (*p* < 0.05, *) in the LL-37-treated groups at concentrations of 10 and 30 μg/mL compared with that in the untreated control group (Figure 6C). Conversely, the magnitude of membrane phospholipid disorder showed no differences compared to that in the untreated control (Figure 6D).

### 3.7. Effects of PTX on Bull Sperm

**Figure 7:**
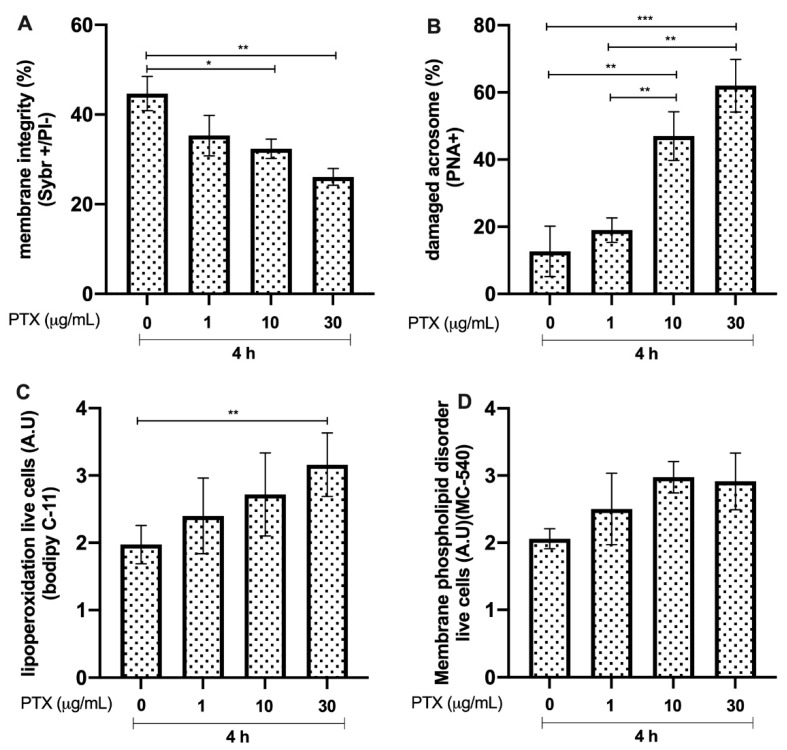
Effect of different concentrations of pentraxin 3 (PTX) on bull sperm. (**A**) Plasma membrane integrity. (**B**) Damaged acrosomes. (**C**) Lipid peroxidation. (**D**) Sperm membrane fluidity. The results are shown as the mean ± standard deviation. The asterisks (*, **, ***) above the bars indicate differences between the groups (*p* < 0.05, *p* < 0.01, and *p* < 0.0001) based on three biological replicates.

Bull sperm membrane integrity after 4 h of incubation with PTX showed a significant reduction in the groups incubated with 10 and 30 μg/mL, compared to that in the control group (*p* < 0.05, * and *p* < 0.01, **, respectively; see Figure 7A). The group incubated with 30 μg/mL PTX showed an increase in acrosome damage, compared to that in the untreated controls and the groups treated with 1 and 10 μg/mL of the protein (*p* < 0.01, **; Figure 7B). Lipid peroxidation increased (*p* < 0.01, **) in the LL-37-treated groups at a concentration of 30 μg/mL, compared to that in the untreated control group (Figure 7C). Conversely, the results of membrane phospholipid disorder showed no differences compared to those in the untreated control (Figure 7D).

## 4. Discussion

During natural coitus and AI in bovines, semen is deposited into the uterus. The NET response usually occurs in utero; is regulated by seminal plasma in horses [16], pigs [17], and donkeys [18]; and has a minor role in the bovine [19]. Irrespective of semen deposition, sperm will migrate through the FRT to fertilise the oocyte; however, a large proportion of spermatozoa remain behind [20]. Sperm migration, as well as excess male gametes, will then result in rapid uterine-derived innate immune reactions, mainly involving PMN-derived effector mechanisms, such as phagocytosis, degranulation, ROS production, and the release of NETs [1,19]. In contrast to NET-mediated cytotoxicity against invasive pathogens [12,13,20,21], very little is known about the adverse cytotoxic effects on sperm, and only a few reports are available on NET-derived cytotoxic effects on human sperm, including altering motility [22] and viability, and resulting in reduced sperm attachment to the zona pellucida [9]. 

SEM analysis confirmed the results of previous work showing that bovine PMN exposed to live spermatozoa resulted in NETs [1]. Moreover, as all involved in NET extrusion were dead, this process can be considered suicidal NETosis (with dead PMN) and not vital NETosis [23]. Interestingly, bovine PMN activation and sperm-induced NETosis can also be increased with the use of different commonly used extenders in bovine AI [19]. Consistent with previous reports in the bovine system, we found fine and thick extracellular NETs entrapping single or various sperm originating from nearby dead PMN. Regarding the bovine sperm-triggered NET phenotypes, the most frequently observed type corresponded to *spr*NETs, which is consistent with previous reports on human sperm [24,25], but not on swine sperm-induced NETosis, where *agg*NETs were the most abundant forms [10]. In terms of excess sperm elimination within the uterus after either AI or natural coitus, suicidal NETosis might contribute more effectively than PMN-mediated sperm phagocytosis or degranulation, as previously postulated [26]. 

Core histones are the most abundant proteins in NETs (comprising 70% of all NET-associated proteins) [27], and NET-derived H2A was recently reported as a potent inducer of epithelial and endothelial cell death in both primary and immortalised cell lines [28]. This damage is mainly caused by a reduction in the inner mitochondrial transmembrane potential, which makes it difficult for the cell to maintain optimal energy levels for its function [29]. In line with this, the current data confirmed this effect, and the cytotoxic damage mediated by H2A in sperm was explained by lipid peroxidation at the membrane level, which led to spermatozoa death along with a loss of acrosome integrity. MPO has been associated with tissue injury, is classified as a hydrogen peroxide oxidoreductase secreted by PMN, and is triggered by DNA and other NET proteins [6]. Classically, it plays a central role in microbial killing and the regulation of oxidative stress at sites of inflammation [30]. In this sense, based on their high MPO enzyme contents, NETs might also contribute via highly oxidative molecules, which could help explain why, in this study, MPO caused severe damage to not only the sperm membrane, but also the acrosome. Surprisingly, the membrane phospholipid disorder, associated with capacitation changes to the spermatozoon, was not affected by the assessed NET-associated proteins included in this study. The current data show that high-level peroxidation in live sperm might be associated with oxidative molecules produced by MPO-like hypochlorite (ClO^−^). Lipid peroxidation, including membrane damage in the presence of NET-derived MPO, could be consistent with previous findings showing progressively decreased motility in swine sperm [17]. In addition, PMN/ELA is secreted by activated granulocytes, and is widely used as a marker of male accessory gland infections [26]. ELA has been described as being involved in the sperm-associated release of NETs in farm animals, such as bovines [1] and pigs, which was shown to be associated with a decrease in total and progressive motility [10,16] and viability, as well as the loss of sperm membrane functions [23]. These findings have also been documented in human sperm [23], thereby confirming our current data based on bovine species. Furthermore, Cat-G, a major serine protease released by activated neutrophils, plays an important role in inflammation through the hydrolysis of host of proteins [31]. In this context, the functional capacity of certain sperm proteins is essential for maintaining the ability of the capacitated spermatozoon to merge successfully with the oocyte [32]. Surprisingly, this protein, similar to the previous proteins, has a negative effect on sperm lipoperoxidation associated with membrane and acrosome damage. This effect on peroxidation was not expected, since it has been reported that the antibacterial action of Cat-G contributes significantly to the non-oxidative antibacterial capacity of neutrophils [33]. LL-37, another important protein in NETs [27], is a cationic host defence peptide that is present in specific granules of neutrophils, and is regulated during inflammation, infection, and injury. Although its direct effect on spermatozoa has not been studied, it has been determined that this peptide induces apoptosis in human and murine airway epithelial cell lines by activating the caspase pathways [34]. Although our experiments do not allow us to associate sperm death with apoptosis-like mechanisms, the damage caused to the sperm membrane by this peptide, together with peroxidative damage, confirms the negative effect on the male gamete. In addition, the finding that PTX is one of the component proteins of NETs and binds to other NET proteins implies its importance in the NET-mediated trapping of sperm [1]. Here, we show, for the first time, that this protein can cause direct damage to sperm, mainly at the membrane, although the role of PTX has been associated with the regulation of NETs in relation to histone cytotoxicity [11]. 

When considering the joint effect of NET-associated proteins, direct NET-mediated damage to the membrane integrity of bacteria [20], fungi [21], and parasites [35] has been described, and this effect has also been reported in human and pig sperm [10,22]. In the spermatozoon, the dynamics of the membrane play a fundamental role in the processes of maturation, capacitation, and fertilisation; however, exposure to NETs can cause a loss of their integrity, as it has been reported for macrophages, dendritic cells, as well as and pig and human spermatozoa [9], as specific damage mediated mainly by a high content and concentration of ELA and Cat-G belonging to these extracellular traps has been described [29]. Moreover, excessive exposure to PMN-derived ROS will lead to oxidative stress with irremediable damage to the sperm membrane [36,37]. Although ROS were not studied in this work, previous studies have linked the mechanism of NETosis to oxidative stress processes in donkey sperm [38]. Here, we confirmed that NET-associated proteins/enzymes/peptides, that is, H2A, MPO, ELA, Cat-G, LL-37, and PTX, have detrimental effects on the membrane of bull spermatozoa, although Cat-G and MPO had more noticeable effects. The greatest cell membrane damage to bovine sperm might occur through membrane lipid peroxidation, as was recently reported, and mainly involving long-chain polyunsaturated fatty acids, docosapentaenoic acid (DPA), and docosahexaenoic acid (DHA) [39]. These findings are relevant for sperm membranes due to their large amounts of DPA and DHA, making them particularly susceptible to damage by this process [39]. The damage mechanism is characterised by autocatalytic oxidative degradation, which occurs throughout the entire lipid bilayer and can consume the membrane partially or totally. This also alters the permeability of the membrane and the acrosome, as we have confirmed in this work for H2A, MPO, ELA, CAT-G, LL-37, and PTX. This oxidative degradation leads to a loss of viability and motility, as well as a reduction in spermatozoon–oocyte fusion ability [22]. In addition, peroxidised lipids are produced, creating new free radicals and a wide variety of cytotoxic compounds [40]. In this context, lipid peroxidation of bull sperm mediated by H2A, MPO, ELA, CAT-G, LL-37, and PTX confirmed that these proteins have direct detrimental effects on the sperm membrane, which could be exacerbated by a high number of other NET-derived proteins [6].

## 5. Conclusions

In conclusion, this study provides valuable information about the cytotoxic effects of the proteins H2A, MPO, ELA, Cat-G, LL-37, and PTX, which are part of NETs on spermatozoa in bovine species, which had a negative effect on membrane and acrosome integrity and lipoperoxidation. These novel data complement with previously NETs-related studies in other species, such as humans and pigs; specifically, when coming into contact with sperm, NETs can play a negative role in reproductive efficiency. However, it is essential to determine the role of sperm-mediated NETs in vivo and to clarify whether certain populations of PMN-releasing NETs, with their battery of cytotoxic proteins, could be responsible for decreasing fertility, which affects the efficiency of the cattle production system.

## Data Availability

The data supporting the findings of this study are available from the corresponding author upon reasonable request.

## Supplementary Materials

The following supporting information can be downloaded at: https://www.mdpi.com/article/10.3390/ani12101308/s1, Figure S1: Gating strategy of flow cytometry of membrane integrity of bovine sperm using Sybr-14/PI. Figure S2: Gating strategy of flow cytometry of damaged acrosome of bovine sperm using PNA/PI. Figure S3: Gating strategy of flow cytometry of lipoperoxidation of bovine sperm using Bodipy C-11/PI. Figure S4: Gating strategy of flow cytometry of phospholipid disorder of bovine sperm using MC-540/Sytox green.
