# Supplementary material for: Adverse Effects of Single Neutrophil Extracellular Trap-Derived Components on Bovine Sperm Function
Source: Animals (Basel). 2022 May 20;12(10):1308. doi: 10.3390/ani12101308 (PMC9138165; doi:10.3390/ani12101308)
Supplement: Supplementary file 1 [file animals-12-01308-s001.zip › animals-1647009-supplementary.pdf]

SUPPLEMENTARY MATERIAL

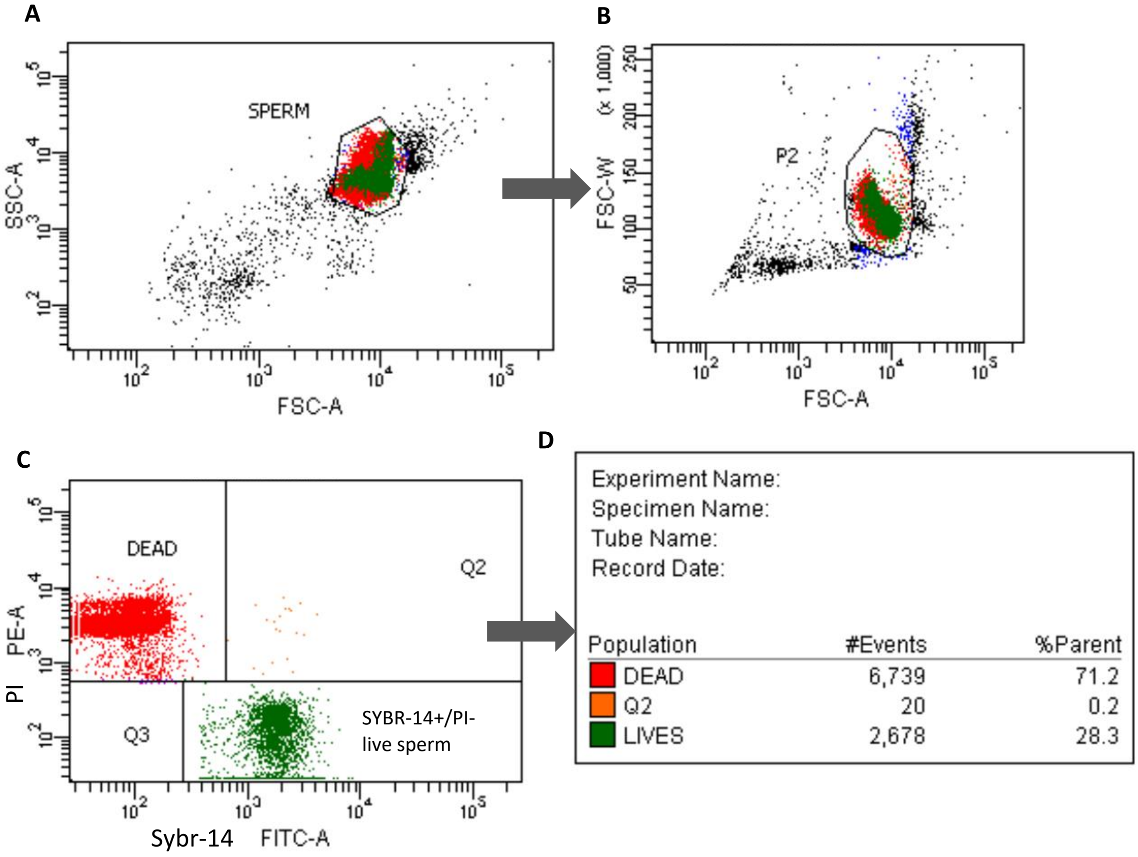

Supplemental figure S1. Gating strategy of flow cytometry of membrane integrity of bovine sperm using Sybr-14/PI. Analysis of representative flow cytometry experiment to determine viability sperm cells. (A) Dot plot of side (SSC-A) versus forward (FSC-A) scatter for each sample. Region of interest according to the gate indicated in (A) to eliminate cellular debris (the areas outlined in black correspond to the gate of bovine sperm population). (B) The forward scatter area (FSC-A) versus forward scatter width (FSC-W) profile was used to exclude cell aggregates and large cells from the analysis. (C) Shows two-dimensional fluorescence plot for Sybr-14 and PI. Four quadrants are indicated, live sperm populations in green (SYBR-14+/PI-) and non-viable sperm populations in red (SYBR-14-/PI+). (D) Shows the quantitative results of the percentages of live and dead sperm population.

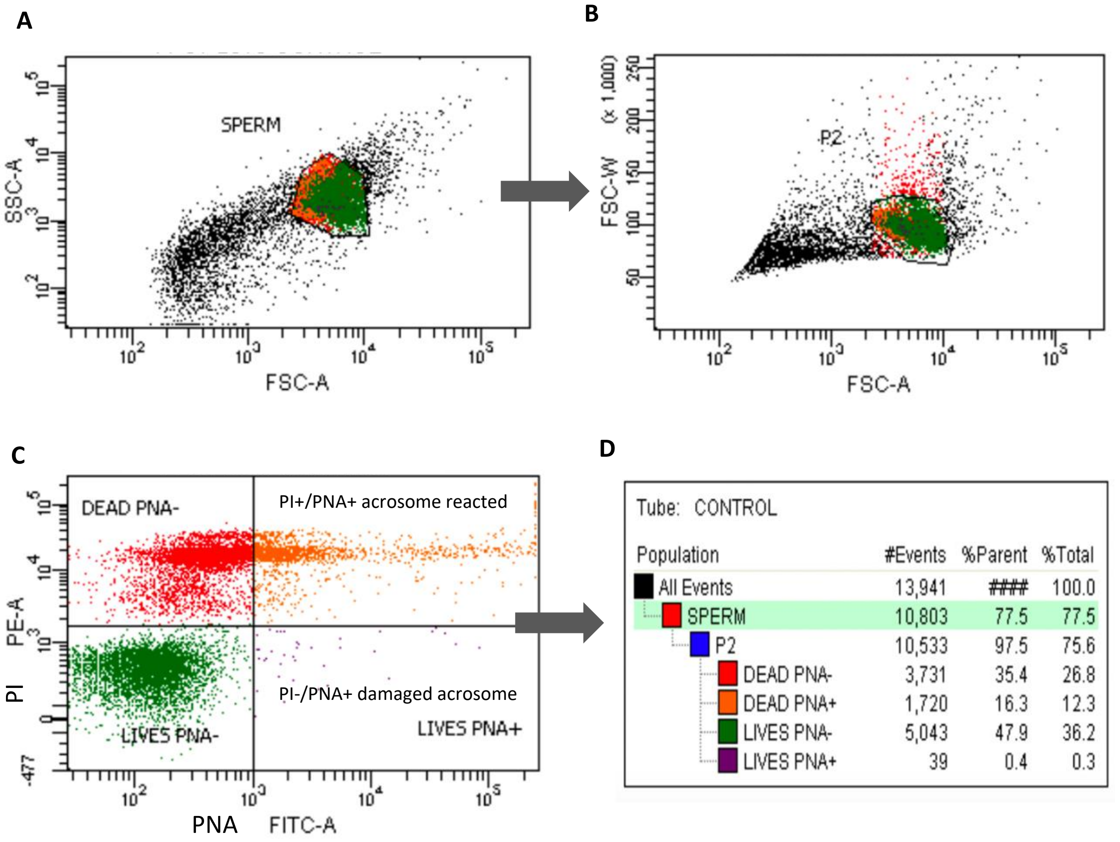

Supplemental figure S2. Gating strategy of flow cytometry of damaged acrosome of bovine sperm using PNA/PI. Analysis of representative flow cytometry experiment to determine acrosome damaged. (A) Dot plot of side (SSC-A) versus forward (FSC-A) scatter for each sample. Region of interest according to the gate indicated in (A) to eliminate cellular debris (the areas outlined in black correspond to the gate of bovine sperm population). (B) The forward scatter area (FSC-A) versus forward scatter width (FSC-W) profile was used to exclude cell aggregates and large cells from the analysis. (C) Shows two-dimensional fluorescence plot for PNA-FITC and PI. Four quadrants are indicated: upper left quadrant (red population) shows non-viable acrosome-intact sperm (PNA-/PI+), upper right quadrant (orange population) show non-viable acrosome-reacted sperm (PNA+/PI+), lower left quadrant (green population) live acrosome-intact sperm (PNA-/PI-) and lower right quadrant contains live spermatozoa with their reacted acrosomes (PNA+/PI-). (D) Shows the quantitative results of the percentages of status of acrosome and live/dead sperm population.

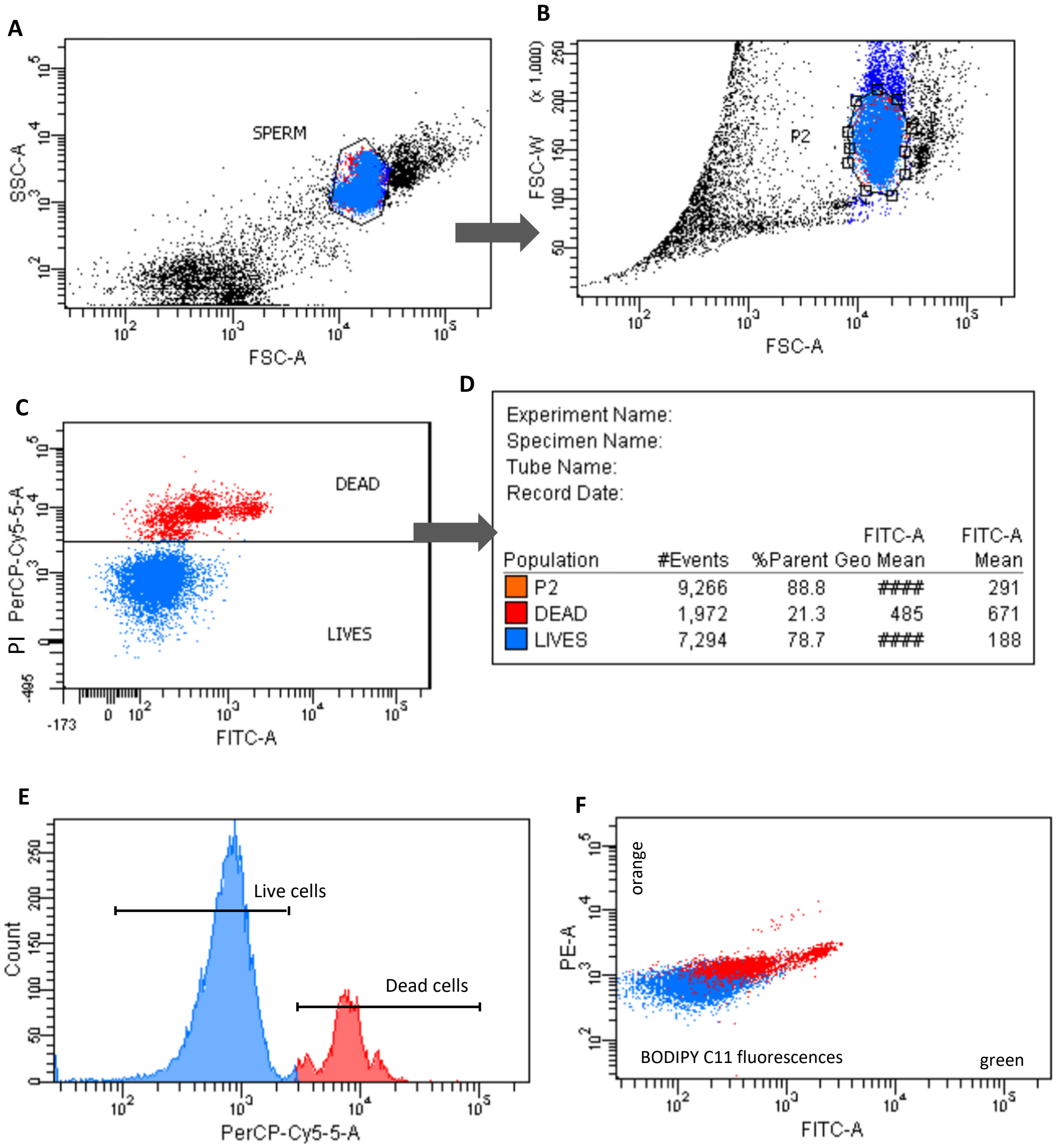

Supplemental figure S3. Gating strategy of flow cytometry of lipoperoxidation of bovine sperm using Bodipy C-11/PI. Analysis of representative flow cytometry experiment to determine lipoperoxidation (LPO) showing selected sperm at 0 time. (A) Dot plot of side (SSC-A) versus forward (FSC-A) scatter for each sample. Region of interest according to the gate indicated in (A) to eliminate cellular debris (the areas outlined in black correspond to the gate of bovine sperm population). (B) The forward scatter area (FSC-A) versus forward scatter width (FSC-W) profile was used to exclude cell aggregates and large cells from the analysis. (C) plot and (E) histogram, Show the separation of live cell (blue) and dead cell (red, marked with PI and read in PerCP-cy5-5 channel). (D) Shows the quantitative results of the percentages and mean of live/dead cells showed MFI of BODIPY C-11 for LPO (FITC, green fluorescence). (F) shows BODIPY fluorescences (orange and green).

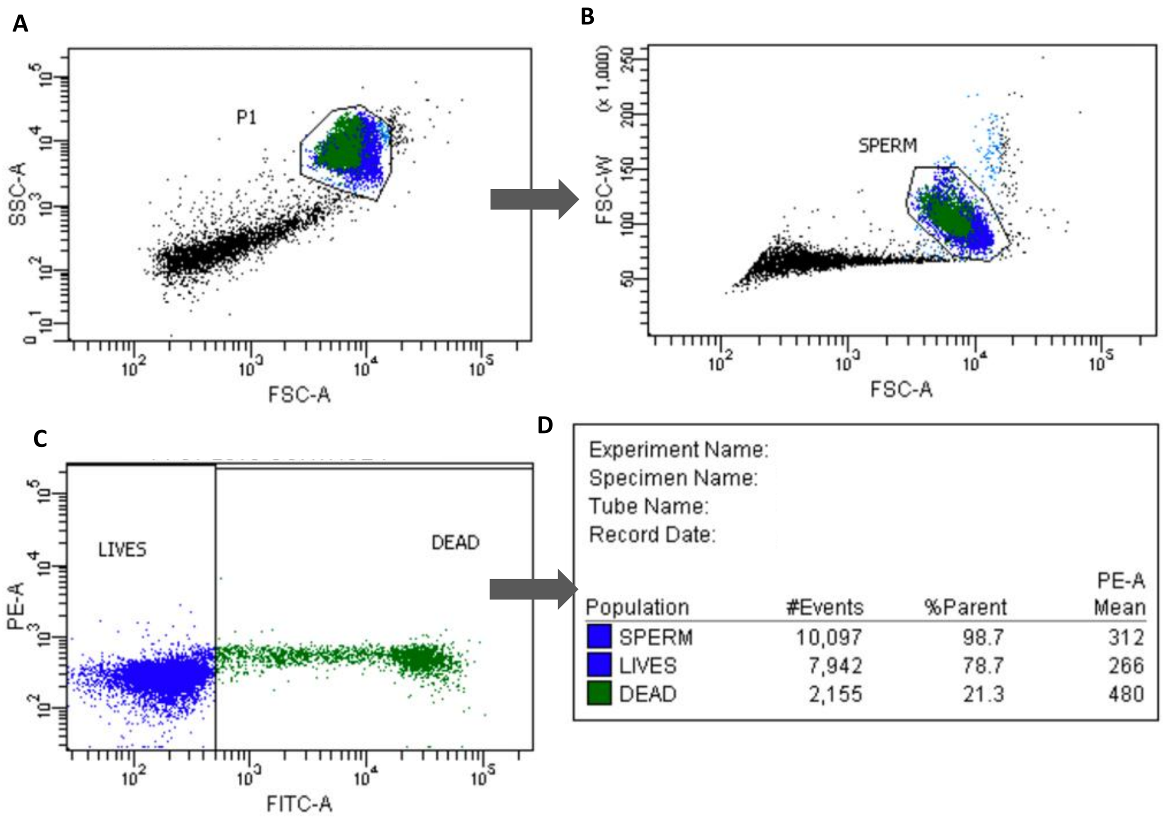

Supplemental figure S4. Gating strategy of flow cytometry of phospholipid disorder of bovine sperm using MC-540/Sytox green. Analysis of representative flow cytometry experiment to determine phospholipid disorder. (A) Dot plot of side (SSC-A) versus forward (FSC-A) scatter for each sample. Region of interest according to the gate indicated in (A) to eliminate cellular debris (the areas outlined in black correspond to the gate of bovine sperm population). (B) The forward scatter area (FSC-A) versus forward scatter width (FSC-W) profile was used to exclude cell aggregates and large cells from the analysis. (C) Shows two-dimensional fluorescence plot for MC-540 and Sytox green. (D) Shows the quantitative results of the percentages and mean of live/dead cells showed MFI of MC-540 for phospholipid disorder (PE, red fluorescence).
